# Supplementary material for: Lactiplantibacillusplantarum ATG-K2 Exerts an Anti-Obesity Effect in High-Fat Diet-Induced Obese Mice by Modulating the Gut Microbiome
Source: Int J Mol Sci. 2021 Nov 23;22(23):12665. doi: 10.3390/ijms222312665 (PMC8657616; doi:10.3390/ijms222312665)
Supplement: Supplementary file 1 [file ijms-22-12665-s001.zip › ijms-1414489-supplementary.pdf]

Supplementary Table S1. Sequences of the primers used for quantitative real-time PCR for in vitro.

**Gene**	**Primer sequence (5′→3′)**
PPARγ	Forward: TTTTCAAGGGTGCCAGTTT<br>Reverse: AATCCTTGGCCCTCTGAGAT
C/EBPα	Forward: TTACAACAGGCCAGGTTTCC<br>Reverse: GGCTGGCGACATACAGTACA
C/EBPβ	Forward: CCAAGAAGACGGTGGACAA<br>Reverse: CAAGTTCCGCAGGGTGCT
SREBP-1c	Forward: TGTTGGCATCCTGCTATCTG<br>Reverse: AGGGAAAGCTTTGGGGTCTA
ACC	Forward: GCGTCGGGTAGATCCAGTT<br>Reverse: CTCAGTGGGGCTTAGCTCTG
FAS	Forward: TTGCTGGCACTACAGAATGC<br>Reverse: AACAGCCTCAGAGCGACAAT
β-actin	Forward: CCACAGCTGAGAGGAAATC<br>Reverse: AAGGAAGGCTGGAAAAGAGC

Supplementary Table S2. Sequences of the primers used for quantitative real-time PCR for in vivo.

**Genes**	**Primer sequence (5′→3′)**
PPARγ	Forward: AGTGGAGACCGCCCAGG<br>Reverse: GCAGCAGGTTGTCTTGGATGT
SERBP1c	Forward: AGCAGCCCCTAGAACAAACAC<br>Reverse: CAGCAGTGAGTCTGCCTTGAT
SCD1	Forward: TCAACTTCACCACGTTCTTCA<br>Reverse: CTCCCGTCTCCAGTTCTCTT
FAS	Forward: CTGGACTCGCTCATGGGTG<br>Reverse: CATTTCCTGAAGTTTCCGCAG
DGAT1	Forward: TCAGATTGAGAAGCGCCTGG<br>Reverse: ACGGAACCCACTGGAGTGAT
PPARα	Forward: GTACGGTGTGTATGAAGCCATCTT<br>Reverse: GCCGTACGCGATCAGCAT
ACO	Forward: GTGCAGCTCAGAGTCTGTCCAA<br>Reverse: TACTGCTGCGTCTGAAAATCCA
CPT1	Forward: TGAGTGGCGTCCTCTTTGG<br>Reverse: TCAGCGAGTAGCGCATAGTCA
UCP1	Forward: CTTTGCCTCACTCAGGATTGG<br>Reverse: ACTGCCACACCTCCAGTCATT
PGC1α	Forward: CCTGAAGCCGGGAGAGAATG<br>Reverse: TAGCCAGCAGAGACTGTGGA
Prdm16	Forward: GAAGTCACAGGAGGACACGG<br>Reverse: CTCGCTCCTCAACACACCTC
ND5	Forward: AGCATTCGGAAGCATCTTTG<br>Reverse: TTGTGAGGACTGGAATGCTG
Dio2	Forward: TTGGGGTAGGGAATGTTGGC<br>Reverse: TCCGTTTCCTCTTTCCGGTG
β-actin	Forward: GGCACCACACYTTCTACAATG<br>Reverse: GGGGTGTTGAAGGTCTCAAAC

Supplementary Figure S1

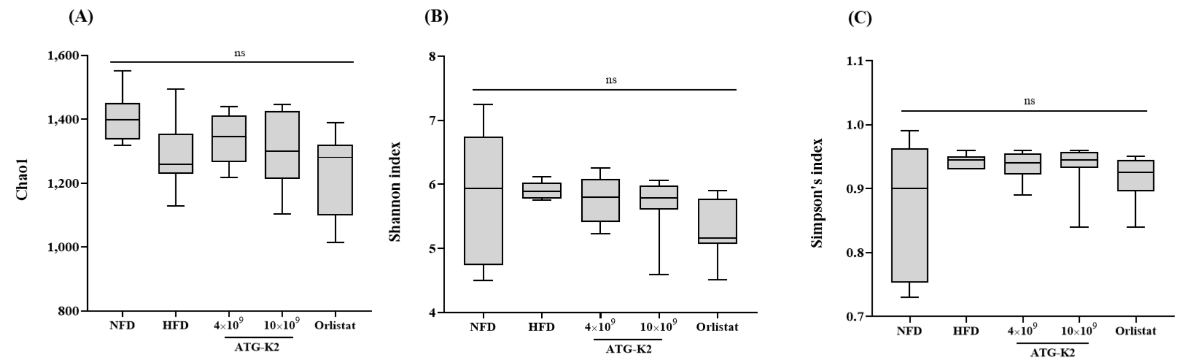

Figure S1. The richness and diversity of *L. plantarum* ATG-K2 in HFD-induced obese mice. (A) Chao 1, (B) Shannon index, and (C) Simpson's index. NFD: Normal fat diet; HFD: High-fat diet; 4 × 10^9, 10 × 10^9: HFD + 4 × 10^9 or 10 × 10^9 CFU/day *L. plantarum* ATG-K2; Orlistat: HFD + Orlistat 15.6 mg/kg. The ends of the whiskers represent the minimum and maximum, the bottom and top of the box are the 1st and 3rd quartiles, and the line within the box is the median.

Supplementary Figure S2

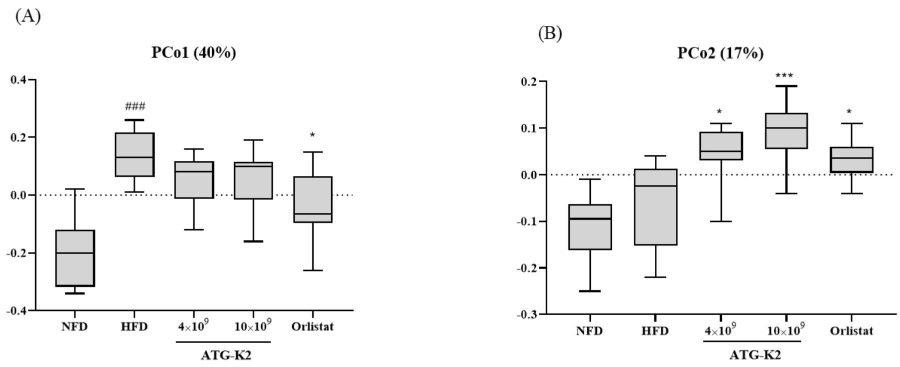

Figure S2. Principal coordinates analysis of *L. plantarum* ATG-K2 in HFD-induced obese mice using UniFrac distance matrix. (A) Examination of the PCo1, (B) Examination of the PCo2. Normal fat diet; HFD: High-fat diet; 4×10^9, 10×10^9: HFD + 4×10^9 or 10×10^9 CFU/day *L. plantarum* ATG-K2; Orlistat: HFD + Orlistat 15.6 mg/kg. $^{\#\#\#}p < 0.005$ vs. NFD group; $^{*}p < 0.05$, $^{**}p < 0.01$, and $^{***}p < 0.005$ vs. HFD group.
